# Supplementary material for: Utility of cochlear perilymph biomarkers for hearing loss - A systematic review
Source: PLoS One. 2025 Oct 17;20(10):e0334351. doi: 10.1371/journal.pone.0334351 (PMC12533882; doi:10.1371/journal.pone.0334351)
Supplement: Attachment — Submitted filename: Final - Response to Reviewers - Perilymph SR - 16.9.2025.docx [file pone.0334351.s002.docx]

Department of Clinical Neurosciences, The University of Cambridge,

Room 605, Clifford Allbutt Building, Cambridge Biomedical Campus,

Hills Road, Cambridge, CB2 0AH, United Kingdom

Tel: +44 (0)7425268324

Email: [stc47@cam.ac.uk](mailto:stc47@cam.ac.uk)

9^th^ September 2025

Associate Professor Toru Miwa,

Academic Editor,

PLOS One

Dear Associate Professor Miwa,

Please find below our responses to the comments and suggestions made by the reviewers for the following manuscript:

Submission ID: *PONE-D-25-17529*

Manuscript Title: “*Utility of cochlear perilymph biomarkers for hearing loss - a systematic review*”

All authors substantially contributed to the production and amendment of the attached manuscript for submission. All authors have reviewed the final version of the manuscript and agree to be accountable for the integrity of the work.

Thank you for considering our revised manuscript for publication in *PLOS One*. We thank you in advance for your time and consideration and look forward to receiving your response when convenient.

Best wishes,

Dr Sita Tarini Clark,

MBChB, BMedSc(Hons), AHFEA

PhD Student, Department of Clinical Neurosciences

University of Cambridge, United Kingdom

**Journal Requirements:**

1. **Please ensure that your manuscript meets PLOS ONE's style requirements, including those for file naming. The PLOS ONE style templates can be found attached.**

We can confirm that our manuscript now meets PLOS ONE’s style requirements, including file names.

2. **We note that the grant information you provided in the ‘Funding Information’ and ‘Financial Disclosure’ sections do not match. When you resubmit, please ensure that you provide the correct grant numbers for the awards you received for your study in the ‘Funding Information’ section.**

We can confirm that the information in the “Funding Information” and “Financial Disclosure” sections of our application match and are correct.

3. **Please note that funding information should not appear in the Acknowledgments section or other areas of your manuscript. We will only publish funding information present in the Funding Statement section of the online submission form. Please remove any funding-related text from the manuscript.**

We apologise for this error and have subsequently removed this information from the title page of our manuscript.

4. **Thank you for stating the following financial disclosure: “Professor Bance and the SENSE Lab are supported by the NIHR Cambridge Biomedical Research Centre (NIHR203312*). The views expressed are those of the authors and not necessarily those of the NIHR or the Department of Health and Social Care. Sita Tarini Clark was funded by the Woolf Fisher Trust, New Zealand, the Cambridge Commonwealth, European, & International Trust, and by Trinity College, University of Cambridge. Nathan Creber was funded by the Garnett Passe and Rodney Williams Memorial Foundation.”. Please state what role the funders took in the study. If the funders had no role, please state: "The funders had no role in study design, data collection and analysis, decision to publish, or preparation of the manuscript." If this statement is not correct you must amend it as needed. Please include this amended Role of Funder statement in your cover letter; we will change the online submission form on your behalf.**

We have now amended our cover letter to include the suggested statement above.

5. **We note that your Data Availability Statement is currently as follows: “All relevant data are within the paper and its Supporting Information files.”. Please confirm at this time whether or not your submission contains all raw data required to replicate the results of your study. Authors must share the “minimal data set” for their submission. PLOS defines the minimal data set to consist of the data required to replicate all study findings reported in the article, as well as related metadata and methods.**

We can confirm that our submission contains the minimal data set / all the raw data required to replicate the results of our study. We have amended our Data Availability Statement on the submission portal accordingly and have also removed this statement from our manuscript file. No supporting files were therefore included in this submission.

6. **If the reviewer comments include a recommendation to cite specific previously published works, please review and evaluate these publications to determine whether they are relevant and should be cited. There is no requirement to cite these works unless the editor has indicated otherwise.**

The editor has not recommended citing any additional work in this manuscript.

7. **Please review your reference list to ensure that it is complete and correct. If you have cited papers that have been retracted, please include the rationale for doing so in the manuscript text, or remove these references and replace them with relevant current references. Any changes to the reference list should be mentioned in the rebuttal letter that accompanies your revised manuscript. If you need to cite a retracted article, indicate the article’s retracted status in the References list and also include a citation and full reference for the retraction notice.**

We can confirm that our reference list is complete and correct. No additional changes were made during this revision.

**Reviewer 1:**

**General comment and recommendation:**

**Authors focus this review on how relevant perilymph biomarkers may be considered for SNHL disorders diagnosis and prognosis, adding results and updates on molecular biology for different SNHL-related disorders published in the last years. This review appears to be well-researched and accurately presented. However, there are some areas where clarity, grammar, and consistency could be improved to enhance readability and comprehension.**

We would like to thank the reviewer for their encouraging comments. Please find a detailed list of our revisions below.

**Comment 1:**

**The term "sacred" might be too informal or subjective for a scientific paper in this journal. Consider using "critical" or "essential".**

Reply 1:

We have amended this sentence accordingly.

Changes in text 1:

*“The live cochlea is a critical and delicate sensory organ with tissue sampling posing a risk to balance and hearing functions.”*

*“This is largely due to the live cochlea being a delicate and relatively inaccessible organ, due to the risk of loss of organ function upon tissue sampling, resulting in hearing and balance compromise.”*

**Comment 2:**

**In the line 148, "Keywords were divided into two groups" could be expanded to explain the rationale behind the grouping of keywords.**

Reply 2:

We have clarified our rationale behind grouping our keywords, as shown below.

Changes in text 2:

*“Keywords were divided into two groups according to biomarker type and hearing loss pathologies.”*

**Comment 3:**

**The inclusion of animal studies in the primary search and their subsequent removal at screening might introduce bias or inconsistencies. It would be better to refine the search strategy to accurately exclude animal studies from the beginning or add a paragraph clarifying how animal vs human studies were reliably identified.**

Reply 3:

We have added a paragraph clarifying how animal versus human studies were reliably identified, as shown below.

Changes in text 3:

*“Excluding animal studies resulted in a minority of the target papers being omitted, as not all human studies were tagged appropriately in the search databases. Therefore, animal studies were included in the primary search and subsequently removed at screening. This was achieved by excluding studies if the population described was clearly animal-based (e.g. rodent, primate, or other non-human models). Where abstracts were unclear, full texts were retrieved to assess the study population; this was performed by two independent reviewers. This process ensured that only human studies relevant to the review question were retained in the final analysis.”*

**Comment 4:**

**In this regard, the use of Google Scholar for identifying target papers before implementing the search might not be comprehensive nor reproducible. It would be beneficial to include other databases or sources to ensure a thorough search. About the type of studies used for this review, the inclusion of correlational studies might need further clarification. It would be beneficial to specify the type of correlational studies included.**

Reply 4:

We included additional databases in our formal search for identifying target papers and have clarified the correlational studies included in this study.

Changes in text 4:

*“In the preliminary stage of this study, Google Scholar was used to identify a broad range of target papers that otherwise may not have been captured by conventional databases. Subsequently, a formal search strategy was performed using only established databases, as listed above.”*

*“Inclusion criteria for this study included: peer-reviewed studies; biomarkers from perilymph associated with hearing loss; human studies; international publications; and correlational studies. Correlational studies included cohort studies in which participants were grouped into different SNHL aetiologies with or without a control group, and associated perilymph biomarkers were identified.”*

**Comment 5:**

**The exclusion criteria list is comprehensive, but it might be helpful to explain the rationale behind excluding certain types of studies, such as "case studies" or "review articles." Same way, in section 2.4, it would be beneficial to explain the rationale behind the selection of specific data points for extraction.**

Reply 5:

Thank you for this recommendation. We have now explained our rationale behind excluding certain study types and selecting specific data points for extraction.

Changes in text 5:

*“Only primary studies were included in this systematic review. Review articles were also excluded to avoid double-counting evidence and introducing bias into the study conclusions. Similarly, case studies were also excluded due to their limited generalisability.”*

*“Some studies included a large volume of screened biomarkers. Those that produced statistically significant results were included in the study analysis.”*

**Comment 6:**

**The exclusion of studies based on sample type at the full-text screening stage might need further clarification. It would be helpful to explain why these sample types were excluded and how this impacts the study's findings.**

Reply 6:

We have included these clarifying details, as shown below.

Changes in text 6:

*“At this stage, the most common reason for exclusion was different sample type (typically serum, CSF, or inner ear tissue). Previously, studies were reliant on these indirect systemic sample types when investigating the cochlear microenvironment. However, due to recent advances in safe intraoperative sampling techniques, researchers are now able to include direct perilymph samples in their analysis. In turn, this review focused on the biomarker profile of these localised perilymph samples specifically, to help inform future studies utilising this novel technique in different hearing pathologies going forward.”*

**Reviewer 2:**

**General comment and recommendation:**

**The systematic review aims to highlight the gap regarding the utility of perilymph biomarkers in SNHL diagnosis, treatment, and prognosis within the current literature. With the availability of perilymph liquid biopsy analyzing tools scientists subsequently have the potential to more accurately characterise inner ear pathologies and provide clinicians with additional diagnostic tools. Therefore, it is essential to "separate the wheat from the chaff" which is to a good extent achieved by this well researched review.**

We would like to thank the reviewer for their supportive comments.

**Reviewer 3:**

**General comment and recommendation:**

**Though multiple studies were reviewed and summarized in the best possible manner but no comparable biomarkers where there in literature. Giving a new classification for future studies doesn`t make a lot of sense without conducting your own study.**

We would like to thank the reviewer for this suggestion and agree with this comment. We have recently secured grant funding for a multi-centre study investigating perilymph biomarkers in hearing loss patients. However, we wanted to first perform a thorough review of the literature to help guide this study and ensure that all relevant perilymph biomarkers were included in our methodology and analysis.
